# Editorial: Parenthood From Biology to Relation. Prevention, Assessment and Interventions for Developmental and Clinical Issues

**DOI:** 10.3389/fpsyg.2018.01042

**Published:** 2018-06-25

**Authors:** Silvia Salcuni, Alessandra Simonelli

**Affiliations:** Dipartimento di Psicologia dello Sviluppo e della Socializzazione Università degli Studi di Padova, Padova, Italy

**Keywords:** parenting, attachment, brain imaging methods, relationships, post-partum depression, parenting measures, treatment outcome

Parenthood represents a fundamental construct that identifies the quality of early adult-infant interactions. In both short and long periods, relationships, as primary interactional experiences, have an essential role in influencing individual's adjustment and psychopathology development during in lifetime. In this scenario, the most important areas of interest and innovation are (a) parents' representation of themselves, both in relation to the child and to their care-giving role; (b) the quality of a couple's relationship, in terms of both conjugal and co-parental bounds, and its influence on the quality of early mother-father-child interactions; (c) the early models of caregiver-baby interaction; (d) the recent approach to the “maternal brain,” that represents the contribution coming from neurosciences, linked to the adult's activation and cerebral functioning processes, in association with the parental role. These data are a starting point for the individuation of functioning mechanisms and developmental trajectories of parenting in groups of adults and babies, belonging to normative populations. At the same time, those studies may provide an important introduction to the detection of critical and/or dysfunctional aspects in population of babies and adults at risk (e.g., preterm babies, adopted children, etc.) or in adults' clinical group (e.g., a depressed parents, addicted parents, etc.) or in children with one or more impaired characteristic (e.g., children with organic diseases, with autistic disorders, etc.).

The present Research Topic puts the attention on these themes, particularly, considering the possible aftermaths that empirical research may have on planning and realizing interventional models to support the parental functioning. We are pleased to publish the contributions of 129 international authors that produced 31 articles, from theoretical review to perspective, from case report to original research issues. The great interest for this topic has made us particularly encouraging in exploring and studying, more and more, the prevention and support to parenting and, also, the specific manners of taking on the therapeutic responsibility of the adult-infant relationship.

The topic focuses both on theoretical and empirical perspective, permitting the individuation of methods of observation and assessment, allowing to plan and realize prevention programs and/or interventions, primarily focused on parental support, both in the early stadium of the child's development and in the long-term period. A particular importance was given to integrative research, which combines different approaches and methods: especially, interactive and/or representational aspects, including data from neuroimaging studies, physiological correlates, and biological data related to parenting. The intercultural aspect care-system between adult(s) and child, as well as review papers on avant-garde themes in this research areas, were also included.

The EBook is organized in sessions, based both on the content and the type of article that we accepted: first, we presented a case report session; then we followed a series of original papers devoted to examining the role of adult attachment in influence and mediated attitudes, symptoms and mental health related to the caregiving system; a series of articles about the neural basis of attachment and the parental brain functioning followed, including both original researches and review articles type; a series of papers considering the “family” viewpoint showed how the family system and the co-parenting can influence both positive and negative children development; and, finally, a new tools validation session, as well as a research on intervention efficacy related to parenting and caregiving system, were presented.

We started the EBook with the important contribution given by three case reports, dedicated to support the very first stages of the mother-infant relationships, respectively within a pediatric primary health care setting (Facchini et al.), enrolling the development of a positive and nurturing dyadic physical contact (Gnazzo et al.), and creating ad hoc attachment-intervention in case of mothers with drug addiction problems (Porreca et al.). All these case reports can be considered as the first step for a more solid research project to assess the efficacy of new tools validation systems (video-feedback in the pediatric system; massages; attachment-related interventions) and to increase dyadic wellbeing throughout positive psychological interventions delivered by practitioners in both clinical and non-clinical but ecological, daily and ordinary caregiving contexts.

A series of original research papers followed, taking into account longitudinally pre-natal and postpartum period with respect to the development of typical vs. atypical parental attachment, feeding practice, and stress characteristics. The role of adult attachment styles in shaping implicit attitudes related to the caregiving system emerged. Maternal attachment remained one of the most important constructs in terms of mediating the psychological relationship with the child (De Carli et al.), in respect to the couple dyadic adjustment (Calvo and Bianco) as well as in relation to child feeding practice and internalizing symptoms (Santona et al.). The attachment pattern was also considered as a mediator in adoptive families, both in the first stage of the adoptive path (Salcuni et al.) and during the adolescence of adoptive children (Pace et al.).

Pre-natal, as well as postpartum period, are important for the well-being of both parents and child. However, in literature, there are a few studies that analyze the relationship between parenting stress, mental health, and dyadic adjustment. The paper we collected with respect to this issue, demonstrated how mental health is an important dimension that mediates the relationship between parenting stress and dyadic adjustment in the transition to parenthood. Effects of symptoms, such as stress, depression and anxiety, were studied during feeding practice in preterm children (Neri et al.; Salvatori et al.), when parents presented eating disorders (Cimino et al.), longitudinally from perinatal to 6 months after birth, in the construction of first time mothers and fathers interaction with the child (Vismara et al.; Mazzeschi et al.), and in relation to dyadic adjustment and parenting stress (Rollè et al.). These findings suggested how much the considered dimensions could be defined as risk factors in the transition to parenthood, and in the very beginning of the emergence of the caregiving system, to establish an emotional bond with their infants.

Recent neuroimaging studies with new mothers and fathers investigated the relationships between parental thoughts/behaviors, neural activation, and infant's developmental outcomes in mothers and fathers. A neuro-imaging mini review of attachment-caregiving system interaction (Lenzi et al.), reported altered activation in limbic and prefrontal areas and in basal ganglia and hypothalamus/pituitary regions. These altered activations are thought to be the neural substrate of the attachment-caregiving systems interaction. Neural and psychological correlates of parental thoughts and actions assessed during the first month postpartum were evaluated showing their effect on child sensitivity (Kim et al.). Results from these neuro-based studies showed how in mothers, anxious thoughts and concerns about the baby were associated with reduced neural responses whereas, in fathers, positive thoughts about parenting were associated with increased neural responses to their own infants. Important papers focused on convergence of psychological, behavioral, epigenetic, and neuroimaging data: Schechter et al. investigated the neuropeptides influences on mothers' PTSD and parenting stress development, and together form a psychobiological signature with direct implications for clinical research on the intergenerational transmission of violent trauma and on motor evoked potential in respect with child gender and crying. The innate predisposition in human adults to respond to infants' signals, in order to satisfy their need and allow them to survive and become young adults capable of taking care of themselves, is well described in a mini review about neural circuits underlying different population parental behavioral responses in front of children's cry (Piallini et al.). The paper by Messina et al. using transcranial magnetic stimulation, highlighted empirically how the brains of adult females appear to be tuned to respond to infant cries with automatic motor excitation. The special issue presented a series of review of existing literature, regarding differences in the parental brain in mammals during carrying practice (Esposito et al.).

In nonclinical families, the co-parenting alliance emerged as a valid mediator between trait anxiety, family system maladjustment, parenting stress (Delvecchio et al.) and the self-perception of parental role (Delvecchio et al.). A higher perception of family maladjustment resulted associated with lower levels of family cohesion and cooperation, and vice versa. The results about co-parenting skills and family adjustment introduced important implications for family studies in Italian culture, and open to comparison with parenting and co-parenting practice in other cultures. Children and adolescents psychopathology in relation to parent characteristics were also analyzed, showing a how caregivers play an essential role in influencing in children eating disorders (Pace et al.) and PTSD and disruptive disorder (Bizzi et al.). Moreover, Cavallina et al. proposed a perspective that underlined how attachment and parental reflective functioning features in child ADHD development.

A methodological session presented the validation of assessment tools. Salcuni et al. presented the Italian validation of the fear survey schedule for children, comparing parents' vs. children's perception of children's fears and confirming how distant these perceptions can be; Skoczen et al. developed and investigated the psychometric properties of the Computerized Family Relations Test (CFRT) for children, demonstrating empirically the consistency of the explored construct. Finally, a valuable review about Emotional Availability Scales and their use, in theory, research and intervention (Saunders et al.) was included.

The last group of original research was devoted to measure effectiveness of clinical intervention in promoting well-being in child-parent relationship (Riva-Crugnola et al.), when postpartum depression is diagnosed (Tambelli et al.), and to explore the association between infant massage practice and mother-infant interaction, in respect with marital adjustment (Porreca et al.).

We hope colleagues can appreciate our topic articles collection and the way in which it contributes to the evidence of the important role of parenting knowledge in psychological assessment and treatment in children.

We would like to thank all the authors and the reviewers who contribute to the present article collection, adding and sharing their knowledge, increasing a better comprehension of the parenting research and clinical field.

## Author contributions

All authors listed have made a substantial, direct and intellectual contribution to the work, and approved it for publication.

### Conflict of interest statement

The authors declare that the research was conducted in the absence of any commercial or financial relationships that could be construed as a potential conflict of interest.

